# M. Tacheron's Anatomico-Pathological Researches in Practical Medicine

**Published:** 1823-12-01

**Authors:** 


### Medico-Chirurgical Review,

AND

### JOURNAL OF MEDICAL SCIENCE.

(Analytical Series.)

"Nec Aranearum textus ideò melior, quia ex se fila fingunt; nec noster vilior, quia ex alienis libamus, ut apes."

Vol. IV.] DECEMBER 1, 1823. [No. 15.

### I.

*Recherches Anatomico-pathologiques sur la Médecine Pratique; ou Recueil d'Observations sur les Maladies Aigues et Chroniques, faites a l'Hospice Clinique interne de la Faculté de Médecine de Paris, et dans les autres Hôpitaux, sous les Yeux de M. M. les Professeurs Corvisart, Leroux, Boyer, Fouquier, Petit, Recamier, Laennec, Jadelot, et autres Médecins recommandables.* Par C. F. Tacheron, M.D. Medecin Civil et Jûdiciare du Onzieme Arrondissement, &c. &c. Three vols. 8vo. Paris, 1823.

"Observatio est filum ad quod dirigi debent Medicorum ratiocinia."
Baglivi.

THE extensive analyses which we have given of the works of Broussais, Lallemand, Parent-Duchatelet, Martinet, and Laennec, in various numbers of this Journal, have been of some service to our brethren on this side of the channel; as presenting them with more elaborate and minute details of pathological investigations than had ever before been conveyed to them through the medium of a review. In this department of medical science, our continental brethren have more zeal, industry, and opportunities than we have—and it has grieved us to see a respected cotemporary of the North set up a claim of superiority on our parts, over the pathological investigations of our neighbours. This is truly ridiculous, and exposes us to the derision of strangers. In therapeutics we have a decided superiority; but no man, not completely blinded by prejudice, could think of claiming the palm of pathology for the profession of these Isles. Even had they the same zeal, industry, and opportunities, still they would not have the *subjects* for anatomico-pathological investigations, in consequence of the active treatment on this, and inert practice on the other side of the channel. But we have said so much on this topic, when reviewing the works abovementioned, that we shall proceed to the work before us, containing an ample *corpus historicum* of cases and dissections.

Pathological anatomy being the science which teaches us the alterations of structure induced by disease, it is evident, that it must be imperfect where the history of the symptoms does not accompany the dissection—for the great object is, to trace the connexion or relation of the *external* phenomena with the changes of organization that are going on *internally*. Without pathological anatomy then, the diagnosis must always be uncertain, and the prognosis hazardous—neither can the therapeutical indications, without it, rest on a solid basis. It confirms or invalidates the diagnosis too hastily established—it teaches the physician what he has to hope and what he has to fear—it prevents him from being lulled into a false security—and leads him (or ought to lead him) to employ early and active measures, so as to give the patient all the advantages that medicine can afford. In fine, he who neglects to examine, on every possible occasion, the bodies of those who die under his care, commits a crime against the living. He is like a mariner who steers without a compass, and who is perpetually led astray by the delusions of the eye. In vain does the physician record the history, note the symptoms, or explore the causes of a disease, if he does not perfect the investigation by making himself acquainted with its actual seat, and the nature of the organic lesions which produce the external phenomena. This last can only be acquired by familiarity with pathological anatomy.

The work before us is constructed on principles that entitle it to confidence—the cases occurring principally in public hospitals, and the notes being taken down in the presence of numerous spectators—"en présence d'un tres grand nombre d'éléves qui tous peuvent répondre de la fidelité que l'on y mettait—de la bonne foi avec laquelle on avouait les malheurs, comme on recontait les succès." To render the work more extensively useful, cases are also selected from the writings of established authors, and the name of each is carefully placed at the head of the case. In this place, we were delighted to learn, that the general administration of the Parisian hospitals had just signed a decree enjoining the annual publication of all important cases. "Elle fait publier tous les ans un ANNUS MEDICUS rempli de faits trés-interressans pour la medecine pratique." By this procedure, (which should long ago have been established) innumerable valuable facts will be rescued from oblivion—pathology will be enriched—therapeutics improved—and knowledge diffused among the profession at large, which would otherwise have been confined to a few individuals. For the honour of this country we hope the example will be followed, not only in the metropolitan, but in the provincial hospitals.

The cases detailed in these volumes have been selected from amidst a vast number—nearly five thousand examples. They have for their object the delineation of those acute and chronic diseases which are daily presented to the eye of the practitioner—and to the contemplation of the young physician, for whom they will serve as a *vade mecum* while pursuing his studies in hospitals and other clinical institutions.

Without attachment to any particular system, our author found it necessary to adopt a certain arrangement, in order to prevent, as much as possible, repetition and confusion. He has also adopted a terse and aphoristic style and language, very unusual in the writings of most of his countrymen.

At the commencement of each order, or class of maladies, a short description is placed, and after the narrative of cases is closed, a few brief reflexions and observations follow.

Our author divides the phlegmasiæ into five orders, according to their seats; *viz.* inflammation of the skin—of the mucous membranes—of the serous membranes—of the parenchyma of the viscera and glands—and finally of the muscular structures.

### ORDER I.—Cutaneous Inflammations.

These would seem to have their seat in the capillary system immediately beneath the epidermis—and, though sometimes the result of local irritation, are far more frequently dependent on affection of a remote organ. They are sometimes sporadic—sometimes epidemic—and very often contagious. They are not always confined to the skin; but are sometimes extended to, or propagated from, the mucous membrane of the stomach and bowels, as has been repeatedly proved by dissection.

Genus I.—*Variola*. This is the product of a peculiar virus—"and is preceded by a febrile movement on the mucous membrane of the stomach." For obvious reasons we shall not touch upon those cases which were not fatal; our object being to ascertain the pathological anatomy of the disease.

*Case* 1. Passing over six cases of distinct small-pox, we come to one of confluent, which proved fatal on the 16th day.

The patient was a youth of 17 years of age, who, on the 7th day, when covered with pustules, the bowels constipated, the pulse strong and frequent, the carotids pulsating violently, the heat of skin intense, &c. had *decoction of bark* in the way of medicine!! To this was added the powder in considerable quantities—but the patient died.

*Dissection.* The sinuses of the dura mater gorged with blood, and some bloody serum at the base of the cranium. *Thorax.*—Two or three ounces (un bon verre) of serum in the cavity of the pericardium—the heart rather larger than natural. The variolous eruptions were very distinctly seen in the mouth, on the velum palati, fauces, and upper part of the larynx. The uvula, velum palati, and trachea were inflamed, the latter containing much mucus. Lungs sound. In the abdomen there were many marks of inflammation.

*Case* 2. This was a baker, 25 years of age, who was taken ill on the 30th of December, and died on the 10th of January. He too had the bark with camphor, &c. and no depletion was used. Dissection, in this case, offered nothing remarkable, except inflammation and pustules on the pharynx.

It is but justice to our author to state that, in his reflections on these two cases, he questions the propriety of exhibiting the bark, and queries whether antiphlogistics and derivatives were not too much neglected?

We need not follow our author in his encomia on vaccination, and his anathemas against the sceptics in its infallibility. These things are old in this country and but new in France. May they never arrive at the same height in the latter, as in the former country!

Genus II.—*Rubeola.* Our author observes that, in mild cases of measles, abstinence, regulated temperature, diluents, and demulcents, are sufficient to conduct to a safe termination; but, when complicated with inflammation of internal organs, we must use depletion, and ought to be cautious how we administer tonics. Of the eight cases of measles detailed by our author, six recovered and two died. Of these last, only one was opened, displaying thoracic inflammation, and enlargement of the spleen and liver. We do not, indeed, see the utility of this section in the work before us.

Genus III.—*Scarlatina.* Only three or four dissections of this disease are given. The first was a youth of nineteen years of age, who was taken ill on the 3d of June with scarlatina, and was treated with emetics, antispasmodics, mindererus, and camphor. He died on the 7th day in high delirium, &c. The head does not appear to have been examined. The interior of the glottis and the intestines were inflamed. No purging medicine appears to have been exhibited.

The second case was a young man of 21 years of age, who was taken ill on the 20th February, and died on the evening of the 26th of the same month. No active treatment was used, and dissection shewed the liver gorged with blood and the intestines inflamed.

The third case was a child of six years, who died on the 12th day of his illness—no active treatment. The right lung was found inflamed, and the left suppurated. The liver was gorged with blood.

The fourth patient, aged ten years, had been epileptic four years. He was taken ill with scarlatina on the 25th April, and died on the 30th of the same month. Orange-flower water formed the principal remedy employed. On dissection, the ventricles of the brain were found deluged with six ounces of water. The mucous membrane of the stomach and bowels was slightly inflamed. One hundred and eighty lombrici, from six to ten inches in length, were found in the jejunum and ileum.

Genus IV.—*Erysipelas;* Genus V.—*Dartre,* we must pass over, as not containing any thing worthy of notice.

ORDER II.—Inflammation of the Mucous Membranes.

We must pass, unnoticed, several genera of this order, as ophthalmia, coryza, otitis, angina tonsillaris, &c. resting a few moments on

*Angina Trachealis.* This inflammation is divided by our author into two varieties—that which is attended with a membranous exudation, and that which is not.

*Case* 1. A woman, 39 years of age, was taken ill on the 13th May, with symptoms of violent inflammation of the upper part of the trachea, but without any thing appearing on inspection of the fauces. Ten leeches, diluents, and an emetic, are the remedies noted down. She died the next day.

*Dissection.* The morbid appearances were bounded to the larynx. The borders of the glottis were thickened and covered with a yellowish and dense layer of exudation—the aperture of the glottis was lessened—the mucous membrane covering the epiglottis was of a livid red colour—the trachea, and branches of the bronchia were filled with mucosities.

*Case* 2. A man, 40 years of age, was exposed to the explosion of a vessel, in which æther was distilling, and, immediately afterwards, was affected with violent headaches, epistaxis, pain in the chest, cough, and hæmoptysis. These symptoms, however, disappeared, but being exposed to a current of cold air when heated, he was seized with sore throat, difficulty of breathing, and pains in his limbs, without fever. When received into the hospital, the tongue appeared red, dry, and rough—pain in his throat—velum palati red—respiration quick, and performed with difficulty—pain in the region of the sternum—no cough. Fifteen leeches to the anus—antispasmodic potion—ptisan. Nothing more, of any consequence, was done, and the patient died three days afterwards.

*Dissection.* The trachea being slit open, there issued a quantity of liquid, which filled it and the bronchia. The mucous membrane was slightly livid, particularly in the neighbourhood of the larynx. There was no other remarkable appearance.

Well may our author observe on this case—"Il est probable que si le malade se fut soumis à un traitment rationnel dès l'origine de cette maladie, on aurait pu espèrer un résultat different." But we do not quite agree with M. Tacheron respecting this *rational treatment.* It is not by "the vapours of warm water and ten or a dozen leeches to the neck,," that we can expect to check acute inflammation of the larynx. General blood-letting should be employed—purging should be used—numerous leeches should be applied to the neck—blisters to the sternum—and antimony with calomel internally exhibited.

*Croup.* Only one fatal case, with dissection, is given by our author. Maria Gambier, three years of age, was seized, on the 10th June, with sore throat, to which no attention was paid. Next day, however, the symptoms were so violent that a physician was called in, and leeches were applied to the neck. Third day, no amelioration of symptoms, and the little patient was bled. In the evening, a blister to the throat, and a purgative glyster. Died this evening.

*Dissection.* The mucous membrane of the trachea red, and lined with a membraniform exudation, thick, and strongly adherent. This false membrane was covered with a puriform matter, and the membrane itself was prolonged into the different ramifications of the bronchia.

GENUS V.—*Hooping Cough.* In this, as in the preceding we shall confine ourselves to the fatal cases accompanied by dissections—pathology being the article of importation on which we set any value in these volumes. We may just premise the cases by remarking, that our author looks upon hooping-cough, in its simple form, as a nervous affection, of an intermitting character; and that it is only dangerous when it terminates in, or is complicated with, inflammation.

*Case* 1. Michael Gallois, three years of age, was taken with hooping-cough in the month of September, immediately after the small-pox. He was sent to the Hopital des Enfans on the 3d of October. The symptoms were not of a formidable description at this time, and the usual routine of ipecacuan, syrups, assafœtida—and (among other means of *cure*) vaccination—were employed. However, the child was getting better, when, on the 3d November, it was seized with convulsions, and died the same night.

*Dissection.* Nothing remarkable in the head. The membrane lining the trachea and bronchia was somewhat redder than natural—the lungs slightly gorged—the liver enlarged —three intus-susceptions in the small intestines—and several contractions of the calibre of the tube in other points, "which shewed that other intus-susceptions were forming." The mucous membrane of the intestines was inflamed in three or four places.

*Case* 2. The following case cannot be called one of hooping-cough; but it is, nevertheless, worthy of record.

Josephine H ——, 30 years of age, subject from youth to nervous affections, entered the hospital on the 12th November, presenting the following symptoms:—emaciation—headache —tongue coated—total loss of voice since the 8th November —breathing embarrassed and quickened—acute pain in the left side of the throat—paroxysms of choaking—dry cough—tumultuous action of the heart—small, quick, and irregular pulse—dyspnœa on going up an ascent—sleeplessness—starting up from short slumbers—hysterical fits of laughing. These symptoms continued, with some variations, till the 16th January, the paroxysms of difficulty of breathing, threatening suffocation, being the prominent and most distressing phenomena. On the last-mentioned day, this poor young woman expired in a paroxysm of most dreadful agony. Most of our readers will be prepared for finding serious organic disease. They will be greatly disappointed.

*Dissection.* The mucous membrane of the pharynx slightly inflamed—that of the larynx and trachea perfectly sound. The lining of the bronchia was red—the lungs rather gorged —the heart somewhat larger than usual—all other parts of the body quite healthy.

"In this case," says our author, "I never saw such paroxysms of menacing strangulation. All the symptoms led us to suppose that there was some tumour pressing upon the trachea, which obstructed the passage of air, and caused such a noise in breathing as was horrible to hear (un sifflement horrible à entendre). At these times, the face presented the appearance of a person just strangled—and yet dissection shewed nothing to account for these dreadful, and ultimately fatal, symptoms." 195.

The above case will call to the minds of several medical men of this metropolis, the case of a young lady at Stoke Newington, (whom we are at this time, 20th July, attending) but whose fate is still uncertain. As it is the most remarkable case we ever witnessed, we shall forbear stating it till the termination is known.

GENUS VI.—*Convulsive Asthma.* Five cases of this disease are described by our author, of whom four recovered—and one died, the dissection of which is given. Our author considers this disease as a kind of hooping-cough affecting particularly irritable adults and old people, consisting of a spasmodic contraction of the muscles of the chest, larynx, and diaphragm, returning at intervals. He is not disposed, therefore, to agree with Rostan and his followers, who look on asthma as a symptom of disease of the heart.

*Case* 1. A man, 64 years of age, had been affected with convulsive asthma for four years. He came into the CHARITE at the conclusion of an attack of bilious fever, and there he died about five weeks afterwards, apparently from debility. He was opened in order to ascertain the state of the lungs. These organs were adherent to the parietes of the chest—they were full of air, but their parenchymatous structure was in a singular state of decay—" état de deperissement singulier." The least pressure burst the air-cells and mashed the substance of the lung. " Il suffisait de les presser sur un point pour rompre les cellules aèriennes, et affaisser leur substance comme du papier mâché." There was no extravasation, nor any other lesion of structure there, or elsewhere.

GENUS VII.—*Pulmonary Catarrh.* Of all the inflammations to which the human frame is subject, there is probably not one of such frequent occurrence as catarrh. The anatomical characters of this phlogosis are well known to be, *redness*, more or less marked—and sometimes a certain degree of *thickening* of the membrane. The bronchial inflammation is accompanied by a secretion of mucus, more abundant than in a state of health, and varying much in its quality in the different stages of the phlogosis. It seldom occupies the whole of the lining of the bronchia, or even of one entire lung. When it does, the disease is serious, and violent fever attends. The causes of this disease are sufficiently well known in this country at least.

The cases where dissection was permitted are small in number, in this section of the work, and will detain us but a very few minutes.

*Case* 1. Guilleminet, aged 36 years, was seized with pulmonary catarrh in the month of January, 1812, which terminated fatally, on the 17th April following, at the end of an epileptic paroxysm, occasioned by a dispute with one of his comrades. It is evident, therefore, that the death was to be attributed to the epilepsy, rather than to the pulmonary affection.

*Dissection.* The vessels of the meninges, and the sinuses of the brain were gorged with blood. Much serum was also effused into the lateral ventricles and at the base of the brain. The right lung was gorged with black blood, and adherent to the costal pleura. The left lung was sound.

*Case* 2. Tranchepain, 52 years of age, was seized with severe pulmonary catarrh in the month of February, and died of the same on the 12th April following.

*Dissection.* The right lung solidly hepatized. When cut into, a puriform matter flowed from several points in the superior portion. The left lung was sound, as were all other parts.

It is a curious pathological fact, which every attentive observer must have repeatedly noticed, that the right lung is infinitely more frequently the seat of disease, especially of this condensation or hepatization, as it is called, than the left. What can be the reason of this ?*

*Case* 3. Barthelet, aged 77, entered the hospital on the 28th March, affected with symptoms of intense pulmonary catarrh. The breathing was laborious and noisy—the cough strong, and recurring in paroxysms of long duration—the expectoration white, and mixed with specks of yellowish matters resembling pus. On the 1st of April, the patient complained of bad taste in his mouth, and pain in his head. From the 4th to the 6th these symptoms increased—the oppression became excessive—the tongue brown—the breathing short and noisy—the pulse small, frequent, and sometimes intermittent—tremors of the limbs, &c. These becoming

* Is it because the liver is opposed to the descent of the diaphragm on the right side, and, consequently, prevents so free an expansion of the right lung, at each inspiration, as on the left side ?

exasperated, there occurred a discharge of pure blood from the intestines, and death took place on the 12th April, 1812.

*Dissection.* The right lung adherent to the costal parietes by ancient attachments, but sound and crepitant in its substance. The left lung sound. The rest of the viscera healthy, except about seven inches of the ileum, just before it arrives at the cœcum, which portion was red, inflamed, and thickened interiorly. It was lined with a substance resembling chocolate.

We believe that there is no inflammation so insidious, at times, as that of the intestines. We lately assisted Mr. Pretty, of Mabledon Place, in opening the body of an old lady who, previous to her death, had not a single symptom of abdominal inflammation; yet the cœcum was found sphacelated, and the peritoneum, in many places, nearly in a similar condition.

*Case* 4. A man, aged 58, had received a fall in the month of August, 1820, which injured the chest, for which he was treated methodically at the hospital, and discharged apparently cured, in a short time. He experienced, however, some symptoms of pulmonic affection till November, 1821, when he was re-admitted, exhibiting the following phenomena. Inclination to lie on his back—sleep disturbed by distressing dreams—too much colour in the face—tongue white—breathing pretty free—some cough, with viscid expectoration—pain in the lower portion of the right side of the chest, where percussion elicited a dull sound—diarrhœa for two days past—pulse frequent. These symptoms went on increasing till the 2d January, 1822, when he died.

*Dissection.* The right lung hepatized, and containing some depôts of purulent matter. The left lung was rather denser than natural. Several portions of intestine inflamed.

Under the head of Hæmoptysis we have a great number of cases, but only one or two dissections. We shall present the reader with one.

*Case.* Mons. R——, had been affected with a catarrhal complaint for some time, when, in crossing the Louvre one day, he was seized with hæmoptysis, and brought up a great quantity of blood. Having reached his home the bleeding stopped; but he continued to lose strength—his breathing was embarrassed and oppressed. He entered the hospital on the 21st July, presenting the following phenomena. Could only sit in bed—breathing short, accompanied by heaving of the sternum—face flushed—lips of a violet colour—pulse feeble, small, and frequent—occasional fits of coughing. Bled to 10 ounces. 22d. The symptoms less distressing—debility rather increased—expectoration thick, copious, yellow, and mixed with blood. 23d. The symptoms all exasperated, and he died in the evening.

*Dissection.* The lungs strongly adherent to the thoracic parietes. The right lung completely hepatised, and gorged with blood. On cutting through the parenchyma, the surfaces presented numerous points of incipient suppuration. The left lung was affected in a similar manner, but not near to such an extent. In several places there were cavities or caverns, the surfaces of which were lined with a matter similar to that which had been discharged by expectoration. The heart was greatly enlarged. No other organic lesion.

Genus VIII.—*Gastritis.* When we reflect on the structure, functions, extreme sensibility, and extensive sympathies of the stomach, we cannot wonder at the frequency of its affections—and especially of those morbid states comprehended under the terms irritation and inflammation.

Gastritis may be occasioned by external injuries; but more frequently by acrid substances taken internally—by cold drink taken too soon after being heated with exercise, or agitated by passion—metastasis of gouty and other inflammations from distant parts—imprudent use of emetics—suppressed perspiration, and various other causes known or unknown. Most commonly, gastritis is complicated with inflammation of the mucous membrane of the intestines—constituting the *gastro-enteritis* of Broussais and his numerous followers—or rather, indeed, the great mass of what were formerly denominated bilious, gastric, and idiopathic fevers. These complications, however, will come under separate consideration, after the subject of simple gastritis is disposed of.

*Case* 1. Chartin, aged 44 years, experienced a chill on the 1st of April, 1811, succeeded by general shivering or rather tremor. The day after, he was feverish, with pain in the chest, and some inclination to vomit. An emetic was taken by the advice of a friend, but without effect. He entered the hospital on the 10th of April, presenting the following symptoms:—disposition to lie on the back—some confusion of intellect—flush on the cheeks—lips dry, and rather inclined to a violet hue—yellowish fur on the middle of the tongue—breath fetid—scanty alvine evacuations—urine scanty and high-coloured—temperature of the surface slightly elevated—breathing difficult and noisy—cough, with mucous expectoration—pains in various parts of the body—great anxiety—pulse small, frequent, and sometimes intermitting—thirst keen—great prostration of strength. On percussion, the right side of the thorax emitted a dull sound—the hypochondria rather full—epigastrium tender on pressure. Venesection to eight ounces—a large blister to the chest—infusion of cinchona. At nine in the evening, relief from the bleeding, but he died a few hours afterwads.

*Dissection.* In the thorax no disease or extravasation. The abdomen was greatly distended: the intestines, in several places, were black, and in appearance sphacelated through all their coats. The stomach was of a dark and violet colour—its coats greatly thickened, indurated, and much increased in weight—the mucous membrane was not only phlogosed, but had blood extravasated into its texture. The inner membrane of the jejunum was inflamed through an extent of five or six inches.

*Case* 2. John Meton, aged 24 years, of a weakly constitution, had been subject, since the age of six years, to vomitings of blood, which became more frequent and severe after having received a blow from a musket on the region of the stomach, in the revolutionary war. A fortnight before his entrance into the hospital, he had experienced a severe attack of headache, accompanied by shivering, and great pain in the stomach, with frequent and violent vomitings. When admitted into the Clinique, he had great pain of head and epigastrium—vomiting of black matters, among which was a proportion of blood of a very fetid odour—the same odour was exhaled from the whole body—cough distressing—chest sounding badly in several places—pulse feeble and frequent. He died soon afterwards.

*Dissection.* The left lung was still a little crepitous, but not so the right—both were gorged with blood and very heavy—when sliced, there exuded from the cut surfaces a sanguineo-purulent matter, without shewing any distinct depôt of pus. The stomach was greatly distended, and its vessels extremely dilated—a stripe of a violet-red colour opposite to the liver, four inches long, and three quarters of an inch in width—on the interior surface, another large inflamed patch, not corresponding with the exterior one. What was most remarkable, was the varicose state of the numerous enlarged vessels of the mucous membrane, from which blood issued copiously by the slightest touch of the scalpel—thus disclosing the source whence came the bloody vomitings to which this young man had been subject since the age of six years. "It is probable, (says the narrator) that the mere presence of food in the stomach was sufficient to cause an effusion of blood from these varicose vessels, when they were in a state of turgescence, and thus produce the hæmatemesis."

*Case* 3. This was a case of poisoning by nitric acid, which a man swallowed on purpose of self-destruction. He lived four days in great torture, and, on dissection, inflammation of the œsophagus and mucous membrane of the stomach was evident. The other viscera sound.

*Case* 4. A widow, aged 27 years, having been affected for some months with a discharge, for which, she learnt that Van Swieten's liquor (liq. oxym. hyd.) was a specific, she procured a bottle of it, and took two or three table-spoonfuls daily, without any other dilution than a small quantity of water. In three or four days, she began to experience colicky pains, which she did not attribute to the proper cause, and continued the medicine, as usual, till the bottle was finished, by which time, to the pains, was superadded a severe diarrhœa, accompanied with burning heat throughout the whole line of the digestive tube, sense of constriction in the pharynx, great difficulty of swallowing, disgust of food, nausea, bad taste in the mouth, thirst, white tongue, pain in the epigastrium, fetid stools, dry skin, acute pain in the heels, pulse feeble and frequent, great prostration of strength, extinction of voice. In this deplorable condition, she entered the hospital on the 26th October, 1819. No proper treatment had been instituted previously.

The symptoms above enumerated, continued to increase in degree of violence till the 18th November, when she expired, vomiting till the last.

*Dissection.* The right lung was sound, the left tuberculated, and some of the tubercles in a state of suppuration. The lower portion of the œsophagus was tinged of a yellow colour, which extended into the stomach. A black fluid was found in this last organ, the pyloric orifice of which was encircled with numerous thickened bands. This thickened condition pervaded the whole extent of the small intestines, there being many black spots and ulcerations about the valvulæ conniventes. The mucous membrane of the stomach and small intestines was soft and pulpy. There were black spots on the peritoneum.

*Case* 5. *Nux vomica.*—A young woman, in consequence of disappointment, determined to take poison, and applied to a chemist, where she procured, under false pretence, about a drachm of nux vomica, which she took in a glass of wine. In a quarter of an hour, she felt uneasiness, with sense of constriction, weight, and heat in the stomach, mouth, and pharynx—painful lassitude of the limbs, followed by convulsive movements and stiffness, especially about the knees. Presently a tetanic stiffness spread itself over the whole frame—the trunk and head were bent backwards—the jaws rigidly locked—the limbs strongly extended—the intellects disturbed. A quantity of milk was exhibited, and much vomiting followed. She was conveyed to the CLINIQUE the same evening, and the following symptoms noted:—Tetanus—convulsive twitchings—crying and laughing—disturbance of the intellects—cheeks flushed—tongue inflamed—thirst urgent—pain in the stomach, and sense of constriction there and in the pharynx—frequent pulse—hot skin. Diluent drinks. On the third day, disappearance of the tetanus—restoration of the intellectual faculties—thirst and vomitings still continued. She gradually recovered.

*Case* 6. *Poisoning by Opium.*—A young man employed in the dispensary of La Charité, determined on self-destruction, and, for that purpose, swallowed about five drachms of the extract of opium, and went to bed. This quantity, however, did not produce the desired effect of ridding him of life, and he, next day, took five drachms more of crude opium. This second dose only provoked some vomiting. On the 3d morning the affair was discovered, and two grains of tartarized antimony were exhibited, which brought off a great quantity of frothy and green matters. Acids were now exhibited—and he slept on the third night, awaking in the morning in a state of delirium. He was examined in the hall of the pharmacy, and appeared pale and wild, with violent headach, tendency to sleep, pulse small, weak, and often irregular, disinclination to answer questions, great debility. Lemonade, cordials. For the next three days, continuation of the same state. The patient complained of severe pain in the stomach—the breathing was short—there was tension of the epigastrium. He dragged out a wretched existence of five days more, and then died, "presque sans agonie."

*Dissection.* Considerable infiltration between the arachnoid and pia mater—some serum in the ventricles and at the base of the brain. The lung of the left side was shrunk and diseased, the cavity of that side being filled with sero-purulent effusion, and the internal parietes lined with false membranes. The heart was sound. There was no perceptible disease in the stomach nor the intestines.

For our own parts, we do not think the patient took one tenth part of the opium he is said to have taken—nor do we think that he died of the effects of the opium at all. There was quite disease enough in the thorax to destroy him.

The next 30 cases are all cancers, open or scirrhous, of the stomach, detailed with endless minuteness and uniformity, quite tiresome to the reader. These we shall pass over entirely. We must also pass over some cases of hematemesis, recorded by our author, as they did not prove fatal, and consequently no light is thrown on the disease by dissection. The treatment pursued was, of course, of the milk and water kind.

Genus IX.—*Phlegmasia Gastro-Intestinalis.* This is the great class which is now making such a noise on the Continent, and which has usurped the place of idiopathic fevers. In the lining membrane of the stomach and bowels, Broussais and his followers place the *fons et origo* of fever in the same confident tone that Dr. Clutterbuck and his followers, in this country, place the throne of fever in the brain and its meninges. Our author is a firm disciple of the Broussaian School, and will swear by the following text of his master:—"*Une phlegmasie locale est toujours le siege primitif de la fièvre qui n'est qu'un phénoméne sympathique ou le resultat d'une douleur transmise au cœur et à tout l'appareil des capillaires sanguins par l'arbre nerveux dont quelques branches font partie de l'organe souffrant.*"

It is of no consequence what the kind of fever may be. If it be contagious, so is this local inflammation. If it be intermittent, so is the local inflammation. If it be endemic, the phlegmasia is endemic. If the fever be epidemic, so is the inflammation. In Egypt the local phlogosis is in the groin—in Jamaica it is in the liver—in Paris it is in the stomach and bowels—in Bridge Street, Blackfriars, it is in the brain. In short, there never was so accommodating a disease as this local inflammation! All the different kinds of fever (improperly so called, say they) are only *shades* (nuances) of local inflammation or irritation.

The first shade, then, (premiere nuance) is a stomach affection, (embarras gastrique,) and "it consists in a collection of bilious secretions, having its seat in the stomach or the duodenum." "Il consiste dans un amas de secretions bilieuses ayant son siége dans l'estomac ou le duodenum." Now "its seat" refers to the collection (amas) and how the Broussaians prove that the seat of bile is in the stomach, we know not. That bile may come from the duodenum into the stomach by the action of vomiting we grant; but that it takes its seat there previously we have no proof whatever. This first shade of phlegmasia is considered by our author as generally symptomatic, and dependent on irritation of the mucous membrane of the stomach and bowels. Fourteen cases are related of this "embarras gastrique," or common bilious disorder, which were all treated by emetics and ptisans with success. But to us it appears rather odd that, if the cause of the bilious collection be *irritation* of the mucous membrane, *emetics* should be employed to remove it. Our author seems aware of this objection, and recommends as an improvement on the emetic plan—"*une methode legerement antiphlogistique et delayante.*" With singular inconsistency M. Tacheron observes, that if in this "first shade of *phlegmasia*" there exist the slightest particle of *phlogosis(!!)* the emetics will do harm! "car s'il y eut existé la moindre phlogose nous aurons observé une augmentation dans les symptoms, au lieu d'une diminution."

To us the simple explanation appears to be this—by numerous causes a mal-action, or mal-secretion is produced in the stomach and bowels, and the consequent depraved secretions produce, in their turn, irritation of the mucous membranes with which they come in contact; and thence radiates the morbid phenomena attendant on this class of complaints. This explanation accords perfectly with the utility both of emetics and purgatives. Remove, by these medicines, the disordered secretions, and you subtract the main source of irritation, besides giving a stimulus to a new and better action of the organs.

Under this head of Embarras Gastrique, or first shade of fever, our author relates two cases of cholera morbus, one of which proved fatal, and presented some peculiarities which render the case worth notice.

A man, 42 years of age, was suddenly seized on the night of the 24th of August, (after having supped heartily on sallad) with violent vomiting and purging of black and green matters, attended with cramps, pain in the epigastrium, and other symptoms of cholera. In thirty-six hours he lost 30 pounds in weight, so great was the evacuation from the system, and so powerfully had the absorbents acted in that time. He was received into hospital on the 26th, his countenance sunk, and complaining of bitter taste in his mouth, headache, anxiety, thirst, constant nausea and vomiting, but no purging. Milk and water practice. 27th. All the symptoms exasperated—great restlessness—abdomen much drawn in and very painful—breathing short and obstructed, but no pain in the chest—pulse small and intermitting. 28th. Delirium added to the other symptoms. 2d September he died.

*Dissection.* A small quantity of serosity in the ventricles and between the coverings of the brain. The pleura was much inflamed, on both sides, with layers of coagulable lymph thrown out by inflammation. The bronchia were filled with mucus. The abdomen prodigiously retracted—the œsophagus, stomach, duodenum, liver and ducts, perfectly healthy. The small intestines remarkable for their state of contraction. They, as well as the peritoneum, appeared of a dark red colour. A considerable portion of ileum was impacted firmly in the pelvis.

The marked pleuritis and the sudden and excessive loss of weight are the two most remarkable circumstances in this case. It is an example, however, proving that great commotions in the functions of vital organs will produce death as effectually as violent inflammation. No one can suppose that the phlogoses described above were the causes of the principal symptoms at the beginning, or even ultimately of death.

### DEUXIEME NUANCE.

We now come to our author's second shade of *gastro-intestinal inflammation*. This affection, he avers, is a necessary consequence of the "EMBARRAS GASTRIQUE," and has its principal seat in the stomach or duodenum—sometimes in the liver and pancreas. "Its symptoms persist *without interruption*, from the invasion till the termination of the disease. It is very common: sometimes epidemic, and greatly varied in character, according to the place and season where it occurs." We do not see how the above description or definition agrees with a passage half a page farther on, where our author states this *phlegmasia* to be susceptible of taking on different types—particularly the *intermittent* type. "Elle est egalement susceptible de prendre différens types—celui d'*intermittent* est le plus fréquent." There were but two desiderata necessary to render the theories both of Broussais and Clutterbuck triumphant. These were the admission, on the part of their adversaries, that inflammation might cease to exist every second day—and that after death there was no necessity to find any trace of the inflammation that destroyed life. The intermittent character of inflammation is boldly proclaimed by Broussais and his disciples—and the non-necessity of finding marks of inflammation in the brain after death is insisted on by Dr. Clutterbuck. Such trifling admissions or indulgences it would be cruel to deny to the founders of an ingenious theory; otherwise we are free to confess that they appear to us to be very serious objections to the respective doctrines of the abovementioned leaders. It is most true that, in fever, the functions of the brain and of the stomach are almost invariably disordered from the beginning. But then, where is the function that is not disordered? If it were insisted on that some one organ or function must be *first* in order of derangement, and we were forced to name that organ, we should undoubtedly say the brain, in preference to the stomach. The various causes of fever must, we imagine, make their primary impressions on the nervous or sentient structure, of whatever part they are applied to, and these impressions must be immediately conveyed to the brain, where it is natural to suppose the first disordered movement obtains. But allowing this to be the case, all the functions become speedily deranged, and the inflammations or disorganizations which we see in the course or at the close of fever, are purely contingent, depending on the predispositions of the parts, or the habits and constitutions of the individuals. The cases brought forward by M. Tacheron himself almost invariably shew head-ache as the first symptom, which is accompanied or followed by the gastric derangement. Not that we deny that affection of the stomach will speedily produce head-ache; but, in fever, it is extremely difficult to say what organ is the first to be disordered in function.

We shall not dwell on this *second shade* of gastro-intestinal phlegmasiæ, as our author has only favoured us with one dissection. Of that case, however, we shall take some notice.

*Case* A young man, ætat. 24, of strong constitution, had been subject, for ten or twelve years, to pain in his head, accompanied by frequent epistaxis. On the 3d of March, 1816, he felt a weight and pain of head, and general lassitude. On the 7th there was much debility, heat, thirst, anorexia, looseness of bowels, with colicky pains, cough, pain in the chest, sleeplessness; of all which symptoms there was an evening exacerbation.

On the 11th he entered the hospital. His face was flushed, lips red, tongue yellow in the middle, thirst urgent, disgust of food, epigastralgia, colicky pains. Eight stools of a bilious character in the last 24 hours. He had a good deal of cough, pulse frequent and hard, great heat, weight and pain in the head. *Venesection to* 8 *ounces*—15 *grains of ipecacuanha and two of emetic tartar.* 12th. The blood was strongly buffed and cupped—the vomiting had been copious —five stools—much pain on pressure in the epigastrium. All these symptoms went on, and nothing was done but administer acidulated whey—and foment the abdomen!! He died, and on dissection some ulcerations were found in the colon—the mesenteric glands inflamed—"and all other organs (the author says) healthy."

Here, then, we have a case of "*gastro-intestinal inflammation,*" without any marks of phlogosis in the stomach or intestines, except the ulcerations in the colon, which were clearly the effect of the dysenteric complaint. As for the treatment—good Heaven protect us from such milk and water practice, either in France or England!

THIRD SHADE.

This is the "gastro-enterite" of Broussais, and the "fièvre adynamique" of Pinel. Our author professes to range himself under no particular banner, but merely to state facts, and allow his readers to judge for themselves.

The disease under consideration very frequently developes itself in people who have recently exchanged a country-life for that of the town, where a less salubrious air, diet, and occupation, contribute to deteriorate the constitution. It is strongly predisposed to, if not actually excited also, by dirtiness, poverty, the miseries of besieged places, the vitiated air of hospitals and dissecting rooms, or any place where there is a too crowded population, the emanations from putrifying animal and vegetable matters, the depressing passions—in short, by the whole range of causes which tend to develop what we designate idiopathic fever.

Its invasion is rarely sudden. It is usually preceded by mal-aise, tremors of the muscles, sense of weakness, defect of appetite and digestion, &c. In some, however, the accession is sudden and more violent, attended by nocturnal exacerbations and daily remissions. The duration of the disease is, on an average, from three to four weeks. In this country, granting that these adynamic or low fevers be the effect of gastro-intestinal phlegmasia, it is certain that the ordinary range of their duration is at least a week less than that which is stated by our author, as observed in France. This must either depend on a difference of disease, of constitution, or of treatment—probably on all three. This species of phlegmasia, our author remarks, may readily complicate itself with other inflammations of particular parts or organs, which, of course, will render the disease more tedious and dangerous. The fatal termination is often determined, he avers, by the use of tonics too early begun, or the omission of proper means at the beginning of the phlegmasia.

We shall pass over 46 cases detailed by our author, because dissection is the only proof we are disposed to accept of from him, of the actual existence of the phlegmasia under consideration.

*Case* I. A shoemaker, 21 years of age, of strong constitution, was taken ill on the 3d of August, 1820, (after having experienced a troublesome cough for a long time) with symptoms of disordered stomach. On the 4th there was debility, with head-ache, thirst, anorexy, and some nausea. A physician prescribed an emetic, which produced violent vomitings of bilious and blackish matters, followed by several loose stools. On the 6th a purgative, and for the next eight or ten days hot sugared wine, (vin chaud sucré.) On the 16th he entered the hospital, presenting the following symptoms, viz.—supination in bed, despondency of countenance—anorexy—prostration of strength—lips chapped and black—tongue red and moist at the edges—respiration rather tight—thirst excessive—seven or eight stools to-day—skin hot and dry—pulse small and weak. *Lemonade—a lavement.* On the 18th and 19th all the symptoms were exasperated, and on the latter day he died. Here our author gravely blames the whole train of bad symptoms, and even death, on the *purgative,* which was administered in the early part of the complaint. Although we are far from agreeing with those who prescribe repeated purgation in dysenteric affections, where, of course, there is actual inflammation or high states of irritation in the mucous membrane of the digestive tube, yet we cannot believe that a single purgative—and particularly a French purgative, at the commencement of a febrile affection, such as that described above, could possibly produce so much mischief as is here laid to its charge. We are far more inclined to place the evil to the account of the hot wine system which was pursued for the eight or ten days succeeding the emetic and cathartic. But we must state the appearances *post mortem.*

*Dissection.* There were two ounces of a reddish effusion under the arachnoid membrane. The inferior lobe of the lungs inflamed and gorged with blood. In the stomach an erosion was found in the great curvature, nearly an inch in diameter, and the parietes were much thinner than natural for a considerable space around this erosion. The duodenum was slightly thickened and inflamed—there were several ulcerations in the mucous membrane of the ileum.

The existence of gastro-intestinal phlegmasia, in this case, we cannot deny; but really we see no proof that it was the primary affection. Two ounces of effusion under the arachnoid afforded sufficient evidence that phlogosis was going forward in the brain, cotemporary, at least, with the abdominal affection. But the following case will place in a still stronger point of view the rage for system which pervades even those who swear they are entirely free from prejudice.

*Case 2.* Julian, aged 17 years, was seized in the beginning of June with cephalalgia, attended with epistaxis of several hours' duration. The second day there was diminution of the cephalalgia, but return of the nasal hæmorrhage. On the succeeding days there were pains in the limbs—bad taste in the mouth—febrile movements in the system. The patient entered the hospital on the 25th of the above month.

The skin appeared of a pallid and yellowish tint—head-ache —tongue furred—respiration a little impeded—pain and tenderness of the epigastrium when pressed—pulse full and quick. Tamarind water and infusions of mild herbs. 26th. Same state. 28th. Great prostration of strength—tongue encrusted—ten or twelve stools. 30th. Pain in the head—oppression—pervigilium—cold chills through the night. 1st. July, same symptoms, and same treatment. 6th. Delirium —constant agitation. Blisters to the legs—decoction of bark. 7th, 8th, 9th, and 10th. Same phenomena. On the last mentioned day, loss of voice—rattles in the throat, and other mortal symptoms. On the 11th, death.

*Dissection.* The vessels of the brain greatly gorged with blood—*the right hemisphere disorganized by a sanguineous infiltration, so that the natural substance of the brain could not be recognized.* Two table spoonsful of effusion at the base of the brain. In the abdomen some small tubercles were observed on the intestine of a reddish colour.

Thus, then, a youth, of 17 years of age, comes into hospital with unequivocal symptoms of cerebral affection, and nasal hæmorrhage. He is treated first with tamarind water, and, as the symptoms increased, with decoction of cinchona. He dies, and one hemisphere of the brain is found completely disorganized (which is a mere bagatelle)—but "des petits tubercles d'une couleur rouge" are found on the intestines—and lo! the great French "doctrine pathologique" is confirmed! Half a brain destroyed sinks into insignificance, when compared with the tubercles on the intestines! Here it is evident that the practice was as injurious as the doctrine was erroneous. There cannot be a doubt that the youth was lost by the "medecine Hippocratique." At all events, no chance of recovery was given to the patient.

*Case 3.* William Loupart, 39 years of age, of strong bilio-sanguine temperament and constitution, was seized with smart diarrhœa on the 15th July, 1817, which lasted twelve days. 27th. General mal-aise, frontal cephalalgia violent, restlessness and pervigilium in the night. No alteration during the following days, although an emetic had been administered. Entered the hospital on the 3d of August, presenting the following phenomena, viz. great pain in the head, especially in the frontal and occipital regions—insomnium—face flushed and burning—tongue red and dry—breath fetid—*no pain on pressure of the epigastrium*—anorexy complete—thirst insatiable, and craving for cold drink—stools scanty and bilious—urine ditto, and high coloured—skin burning hot, and dry—pulse full and quick—general feeling of fatigue. 4th. There was an exasperation of the symptoms this evening. *Edulcorated whey—two demi-lavements.* During the two following days no change in the symptoms. *Infusion of cinchona—camphor and nitre—blisters to the legs.* On the 8th of August, (12th day of the disease,) there was constipation, with inclination to vomit, somnolency, and slight convulsive movements. 9th August. The face more flushed than ever—cephalalgia exceedingly intense—the look fixed and animated—" in fine, all the symptoms of cerebral congestion." *Ten leeches behind the ears, sinapisms to the feet.* These measures, puny as they were, produced a mitigation of the symptoms. In this state the patient continued till the 17th of August, when a convulsive cough came on that threatened suffocation, but which was moderated by emulsions, &c. He went on again, without any remarkable change, till the 37th day of the disease, when the patient complained of sharp pain in the epigastric region, aggravated by pressure. *Eighteen leeches to the anus—warm fomentations to the abdomen.* Next day leeches (ten in number) were applied to the hypogastrium—and considerable abatement of all the symptoms followed for several days. But a relapse took place on the 42d day of the disease, and all the bad symptoms returned, accompanied by violent delirium, which lasted till death, on the 52d day of the malady.

*Dissection.* A considerable quantity of serous effusion into all the cavities of the brain, and between the membranes. The pericardium and bags of the pleura also contained large quantities of serous infiltration. The abominal viscera appeared perfectly sound—yet, on attentive examination, some marks of inflammation, and even small ulcerations were observed on the mucous membrane of the stomach and intestines.

It is really astonishing that any man, not completely warped by the most inveterate prejudice, could think of putting this case down as an example of gastro-intestinal phlegmasia. Of all the marks of local inflammation left after death—of all the symptoms indicating topical phlegmasia during life, those appertaining to gastric or intestinal phlogosis are *least* evident. And yet they are placed at the head of the list, and the disease acquires " a local habitation and a name" upon such a frail and insecure basis! These cases, however, are very valuable in various points of view, as our readers will very readily appreciate.

*Case* 4. Stephen Christian, 22 years of age, strong constitution, was seized on the 22d August, 1817, without any evident cause or precursory phenomena, with shiverings and tremors, accompanied by cephalalgia, and followed by great heat of skin, and intense pyrexia. *Hot sugared wine* (vin chaud bien sucré.) Exasperation of the symptoms. During the three following days, bouillie, *and half a bottle of hot wine night and morning!* On the 6th of August, four grains of emetic tartar. Much vomiting and purging. On the same day he entered the hospital, presenting the following symptoms, viz. bad taste in the mouth—head-ache—insomnium—red spot on each cheek—lips dry, and tongue covered with a yellowish crust—breath fetid—some cough—anorexia—much thirst—skin yellowish—pulse small, frequent, and rather irregular.

7th August. Face flushed—eyes watery—head-ache intense—some pain on pressing the region of the liver—restlessness. *Infusion of bark.* These symptoms continued gradually increasing, and he died on the 13th of the same month.

*Dissection.* The brain was black with congested blood—the ventricles and the cellular tissue of the arachnoid contained a great quantity of serous effusion. The peritoneum. epiploon, and mesentery, were of a redder colour than natural—some portions of the mucous membraue of the intestines reddened and much injected, but not disorganized in structure.

We think there can be but one opinion on this side of the channel respecting the bad treatment in this case, and the want of any thing like evidence as to the priority of gastro-intestinal inflammation as the cause and essence of the disease.

### FOURTH SHADE, OR "FIEVRES ATAXIQUES." BY M. PINEL.*

If, in the former shades, we have shewn by the dissections and symptoms recorded by the author himself, that the brain was full as much implicated in the fever as the stomach or bowels, it is still more evident in the class of fevers now under consideration. In fact, the author balanced in his own mind, for some time, whether he would not treat of them under the head of "*inflammation of the membranes of the brain.*" Broussais, too, appears to be *hedging* a little; for he now introduces into his new nomenclature a class of fevers entitled —"gastro-enterites et *encephaliques*"—the policy of which is sufficiently obvious. But still the principle is too narrow.

---

* *Adynamique* fevers are those where there is much muscular debility, as the name imports. *Ataxique* are those fevers which are considered *malignant* or irregular in their characters.

Observation will prove that there is no part of the body insusceptible of inflammation in fevers; and, on the other hand, that there is no structure exclusively the seat of phlogosis in this class of maladies. We will go still farther, and maintain our original opinions, that inflammation is not essential to fever at all—that it is not necessarily the first link or cause of the disease; but merely an epiphenomenon or casual occurrence in the course of the fever.

This ataxic fever is stated to be preceded by various phenomena, such as head-ache, somnolency, agitation, despondency, faintings, lassitude, and, in most cases, with symptoms of gastritis. Its duration is from two to four weeks. "In its *intermitting* form the danger is so great that we are obliged to suppress it, or change its character." Death is considered to result, in general, from the abdominal lesions. The convalescence is long and seldom perfect.

Passing over 26 cases of this fever which ended favorably, we shall extract, as an example, a single case where the dissection is appended.

*Case.* Fleuret, aged 30 years, entered the Clinique on the 20th October, 1818, presenting the following symptoms—cephalalgia intense—violent delirium—pupils dilated—cheeks a little flushed—tongue white in the middle and dry—respiration somewhat embarrassed—some pain in the right side of the chest—tenderness in the epigastrium on pressure—thirst—no appetite—some diarrhœa—pulse small and frequent—*whey and rice water—ten grains of camphor*. No more statement is made till the 31st, when blood is taken from the foot, and blisters applied to the lower extremities. On the 1st of November the agitation was extreme and constant. On the 2d, two leeches were applied to the jugulars—ice to the head—fresh sinapisms to the feet. 3d. and 5th. Diminution of the symptoms. 6th. Great delirium—face red—eyes watering—conjunctiva inflamed—tongue black—abdomen painful. *Tonic fomentations to the abdomen—lavement of camphor and cinchona*. These symptoms and this kind of treatment went on till the 14th of December, when the patient died.

*Dissection.* Brain extremely soft—ventricles full of water—thoracic organs sound—mucous membrane of the stomach inflamed—some small ulcerations in the lining membrane of the intestines.

We have been absolutely astonished that, so late as the year 1818, when the doctrines of Broussais were at their height, a man presenting such unequivocal symptoms of congestions or inflammations in various organs, should have been treated in the manner detailed above, in a public hospital of Paris. Of what use are these grand pathological doctrines to their professors? They see the traces of inflammation after death—they record their symptoms during the progress of the disease—they affirm that these inflammatory lesions are the cause of the disease and the cause of the fatal termination—and yet, strange to say, they take not a single step to make their practice quadrate with their doctrines! It is, in fact, those only who look upon inflammation as a contingency in fever who are judicious practitioners. When they see the symptoms indicating local inflammation, they treat them accordingly—and their general plan of treatment is to prevent, if possible, such local inflammations taking place at all. The numerous cases and dissections which follow are so near a-kin to that just now detailed, that we need not analyze any more. In a future number we shall probably take up the phlogoses of the *serous* tissues, and present our readers with some curious specimens of pathology and practice.

